# The comparison of remimazolam and midazolam for the sedation of gastrointestinal endoscopy: a meta-analysis of randomized controlled studies

**DOI:** 10.4314/ahs.v22i2.44

**Published:** 2022-06

**Authors:** Lin Zhang, Chun Li, Chuncheng Zhao, Yulai You, Jian Xu

**Affiliations:** 1 Department of geriatrics, Jiangjin District Central Hospital, Chongqing; 2 Department of Neurology, Jiangjin District Central Hospital, Chongqing

**Keywords:** Remimazolam, midazolam, gastrointestinal endoscopy, meta-analysis

## Abstract

**Introduction:**

Remimazolam and midazolam are used for the sedation of gastrointestinal endoscopy, but their efficacy remains controversial. We conduct a systematic review and meta-analysis to compare the sedation of remimazolam with midazolam for gastrointestinal endoscopy.

**Methods:**

PubMed, Embase, and the Cochrane Central Register of Controlled Trials were searched. Randomized controlled trials (RCTs) assessing the influence of remimazolam versus midazolam on gastrointestinal endoscopy were included. Two investigators independently have searched articles, extracted data, and assessed the quality of included studies. This meta-analysis was performed using the random-effect model.

**Results:**

Three RCTs involving 528 patients were included in the meta-analysis. Compared with midazolam for gastrointestinal endoscopy, remimazolam was associated with higher procedure success (OR=9.78; 95% CI=1.48 to 64.71; P=0.02), lower need for rescue medication (OR=0.09; 95% CI=0.01 to 0.80; P=0.03), shorter total recall (Std. MD=0.93; 95% CI=0.15 to 1.72; P=0.02) and delayed recall (Std. MD=0.44; 95% CI=0.05 to 0.83; P=0.03), reduced incidence of hypotenson (OR=0.39; 95% CI=0.25 to 0.62; P<0.0001) and adverse events (OR=0.36; 95% CI=0.17 to 0.79; P=0.01), but had no obvious influence on fully alert (Std. MD=-0.75; 95% CI=-1.58 to 0.08; P=0.08).

**Conclusions:**

Remimazolam demonstrated better efficacy and safety for the sedation of gastrointestinal endoscopy compared to midazolam.

## Introduction

The sedation for gastrointestinal endoscopy is widely obtained by the benzodiazepine^1^–^3^. One kind of benzodiazepies, midazolam is the most commonly used drug for the sedation of gastrointestinal endoscopy, closed reductions of long-bone fractures, and reductions of dislocations etc^4^–^6^. However, current benzodiazepines have two main disadvantages. Firstly, they provide no analgesia, but residual sedative effects are obtained beyond the duration of the procedure. Secondly, the half-life of many benzodiazepines is approximately 1.8 to 6.4 hours, which results in a longer and less predictable recovery from sedation^7^–^9^. Remimazolam is a new benzodiazepine, and designed as a short-acting drug for intravenous sedation for limited duration procedures such as gastrointestinal endoscopy^10^,^11^. Remimazolam can be raidly hydrolyzed in the body by ubiquitous tissue esterases to an inactive carboxylic acid metabolite^12^. In the first-in-humans phase I trial, remimazolam was found to safely and rapidly induce sedation after a single bolus administration in adults, and the peak effect of sedation could be obtained in approximately 1 to 4 minutes after the start of the infusion. The depth and duration of sedation was sufficient at doses between 0.10 and 0.20 mg/kg. Offset of sedation with remimazolam was obtained between 10 and 20 minutes^13^.

Remimazolam was studied in phase II and III studies which were performed in USA^11^, ^14^, ^15^. Several studies have investigated the efficacy of remimazolam versus midazolam for the sedation of gastrointestinal endoscopy, but the results were conflicting^11^, ^14^, ^15^. We therefore conducted a systematic review and meta-analysis of RCTs to compare the efficacy and safety of remimazolam versus midazolam for the sedation of gastrointestinal endoscopy.

## Materials and methods

Ethical approval and patient consent were not required since this was a systematic review and meta-analysis of previously published studies. The systematic review and meta-analysis were conducted and reported in adherence to PRISMA (Preferred Reporting Items for Systematic Reviews and Meta-Analyses)^16^.

### Search strategy and study selection

Two investigators have independently searched the following databases (inception to July 2019): PubMed, Embase, and the Cochrane Register of Controlled Trials. The electronic search strategy was performed using with the following keywords: remimazolam, and midazolam, and gastrointestinal endoscopy or colonoscopy or gastroscopy. We also checked the reference lists of the screened full-text studies to identify other potentially eligible trials. The following inclusive selection criteria were applied: patients underwent gastrointestinal endoscopy; intervention treatments were remimazolam versus midazolam; study design was RCT; Aged≥18; Body mass index≤40 kg/m^2^; American Society of Anesthesiologists (ASA) physical status score were evaluated. Only RCTs were included in order to reduce the bias.

### Data extraction and outcome measures

We used a piloted data-extraction sheet, which covers the following information: first author, number of patients, age, male, weight, and detail methods in two groups. Data were extracted independently by two investigators, and discrepancies were resolved by consensus. We contacted the corresponding author to obtain the data when necessary.

The primary outcome was procedure success which was defined as (1) Modified Observer's Assessment of Alertness/Sedation (MOAA/S)≤4 on 3 consecutive measurements taken every minute, (2) completion of the procedure, (3) no requirement for an alternative and/or rescue sedative, and (4) no manual or mechanical ventilation. Secondary outcomes included rescue medication (representing that sedative rescue medication was needed if adequate sedation was not sufficiently obtained), total recall (indicating the patient's ability to learn new information), delayed recall (representing patient's ability to memorize new information), fully alert (indicating 3 consecutive MOAA/S scores of 5), hypotension (suggesting systolic blood pressure≤80 mm Hg), and adverse events.

### Assessment for risk of bias

The risk of bias tool was used to assess the quality of individual studies in accordance with the Cochrane Handbook for Systematic Reviews of Interventions^17^, and the following sources of bias were considered: selection bias, performance bias, attrition bias, detection bias, reporting bias, and other potential sources of bias. The overall risk of bias for each study was evaluated and rated: low, when the risk of bias was low in all key domains; unclear, when the risk of bias was low or unclear in all key domains; and high, when the risk of bias was high in one or more key domains^18^. Two investigators independently searched articles, extracted data, and assessed the quality of included studies. Any discrepancy was solved by consensus.

### Statistical analysis

We estimated standard mean difference (SMD) with 95% confidence intervals (CI) for continuous outcomes (, total recall, delayed recall, and fully alert) and odds ratio (OR) with 95% CI for dichotomous outcomes (procedure success, rescue medication, hypotension and adverse events). A random-effects model was used regardless of heterogeneity.

Heterogeneity was assessed using the I2 index and Cochran's Q test. I2 index values lower than 25% indicated low, 26–50% moderate and more than 50% high degree of heterogeneity, and Cochran's Q statistic p<0.05 were considered indicators for significant heterogeneity. Whenever significant heterogeneity was present, we searched for potential sources of heterogeneity. Sensitivity analysis was performed to detect the influence of a single study on the overall estimate via omitting one study in turn when necessary. Owing to the limited number (<10) of included studies, publication bias was not assessed. Results were considered as statistically significant for P <0.05. All statistical analyses were performed using Review Manager Version 5.3 (The Cochrane Collaboration, Software Update, Oxford, UK).

### Quality of evidence

The quality of evidence for each outcome was evaluated based on the methodological quality and the confidence in the results, and it was assessed by GRADE recommendations as high quality, moderate quality, low quality, or very low quality^19^.

## Results

### Literature search, study characteristics and quality assessment

**Figure 1:**
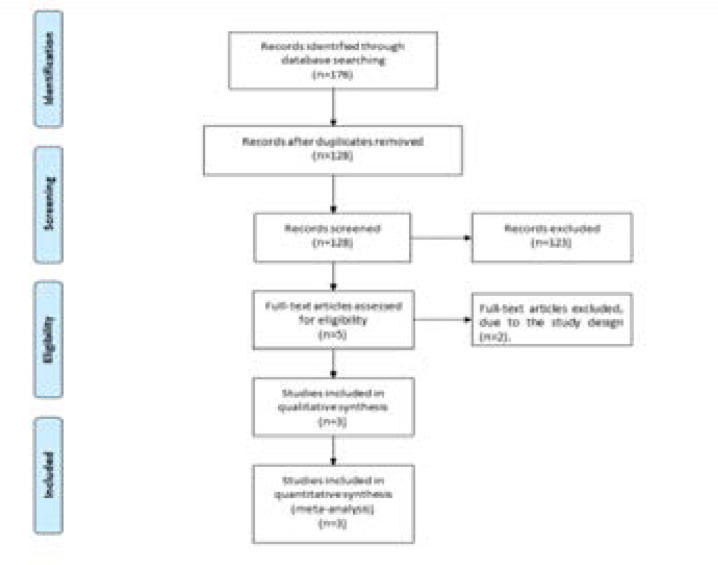
Flow diagram of study searching and selection process.

A detailed flowchart of the search and selection results was shown in Figure 1. 176 publications were searched after the initial search of databases. 48 duplicates and 123 papers after checking the titles/abstracts were excluded. Two studies were removed because of the study design and three RCTs were ultimately included in the meta-analysis^11^, ^14^, ^15^.

The main characteristics of the three included RCTs were presented in Table 1. The three studies were published between 2015 and 2018, and sample sizes ranged from 50 to 398 with a total of 528, and detail methods of remimazolam and midazolam were different in each RCT. Sample size calculations for the study conducted by Rex et al.^11^ were based on the following assumptions: For a 1-sided type I error rate of 0.025 and a target power of 90%, the assumption of a success rate of 30% for the placebo group and 90% for the remimazolam group led to sample sizes of 15 patients per treatment group. However, 300 patients were required for the remimazolam group in order to reach an appropriate size for the safety database. The midazolam group was included for assay sensitivity and set at 100 patients.

**Table 1:** Characteristics of included studies

NO. Author		Total sample size	Remimazolam group					Midazolam group					Jada scores
			Number	Age (years)	Male (n)	Weight (kg)	Methods	Number	Age (years)	Male (n)	Weight (kg)	Methods	
1	Rex 2018	398	296	54.4±10.12	147	83.2±17.39	an initial single intravenous dose of remimazolam 5.0 mg, maintained by injection of further top-up doses of remimazolam 2.5 mg	102	55.6±10.15	46	81.9±16.24	3 doses in any 12- minute window(as per the midazolam package insert: 1.75mg initial/1 mg top-up dose for a patient aged <60 years, and 1.0 mg/0.5 mg for those aged 60 years, debilitated, or chronically ill)	4
2	Pambianco 2016	80	40	55	-	-	remimazolam (5.0 mg) for the induction of sedation, maintained by 3.0 mg	40	55	-	-	midazolam (2.5 mg) for the induction of sedation, maintained by 1.0 mg	3
3	Borkett 2015	50	25	41	-	-	a single dose of remimazolam 0.20 mg/kg	25	41	-	-	a single dose of midazolam 0.075 mg/kg	4

Among the three RCTs, three studies reported procedure success and rescue medication^11^, ^14^, ^15^, two studies reported total recall and delayed recall^14^, ^15^, two studies reported fully alert, hypotension and adverse events^11^, ^15^.

### Assessment of risk of bias

**Figure 2:**
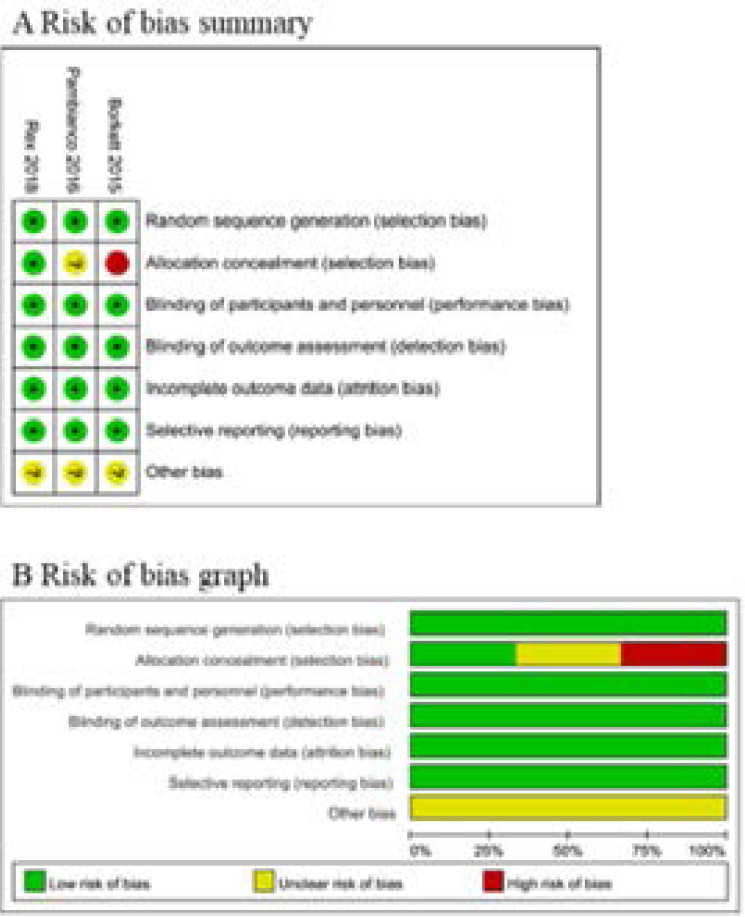
Forest plot for the meta-analysis of procedure success.

Risk of bias analysis (Figure 2) showed that one study had high risk of allocation concealment due to the open label of midazolam^15^, but all RCTs generally had high quality.

### Primary outcome: procedure success

**Figure 3:**
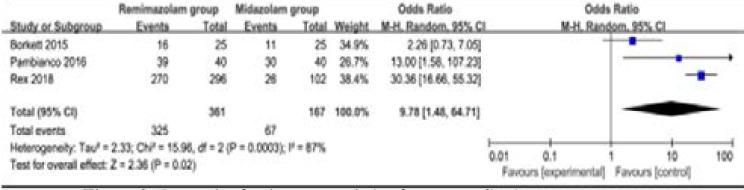
Forest plot for the meta-analysis of rescue medication.

This outcome data was analyzed with the random-effects model, and the pooled estimate of the three included RCTs^11^, ^14^, ^15^ suggested that remimazolam results in higher procedure success for gastrointestinal endoscopy than midazolam (very low quality, n=528; OR=9.78; 95% CI=1.48 to 64.71; P=0.02), with significant heterogeneity among the studies (Chi2=15.96, P=0.0003, Figure 3).

### Sensitivity analysis

Significant heterogeneity was observed among the included studies for the procedure success. As shown in Figure 3, the study conducted by Borkett showed results that were almost out of range of the others and probably contributed to the heterogeneity^15^. After excluding this study, the results suggested that compared with midazolam for gastrointestinal endoscopy, remimazolam was associated with higher procedure success (OR=28.49; 95% CI=15.99 to 50.74; P<0.00001, two RCTs included11, 14), and no heterogeneity remained (Chi2=0.60, P=0.44).

### Secondary outcomes

**Figure 4:**
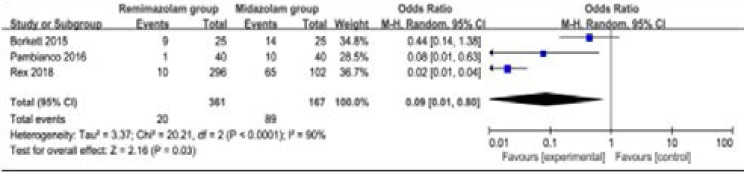
Forest plot for the meta-analysis of total recall.

**Figure 5:**
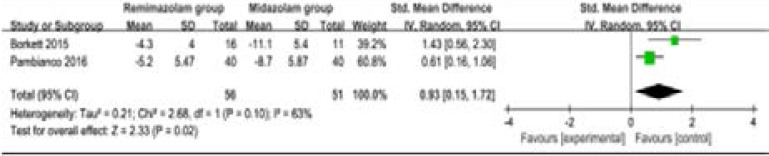
Forest plot for the meta-analysis of delayed recall.

**Figure 6:**
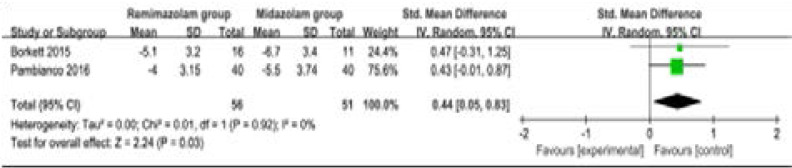
Forest plot for the meta-analysis of fully alert.

**Figure 7:**
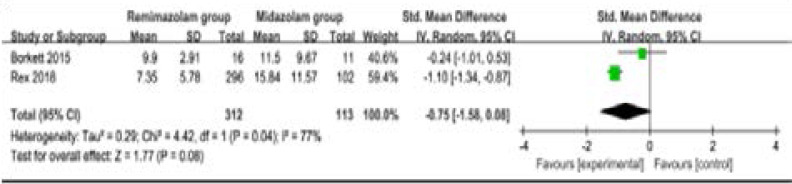
Forest plot for the meta-analysis of hypotension.

**Figure 8:**
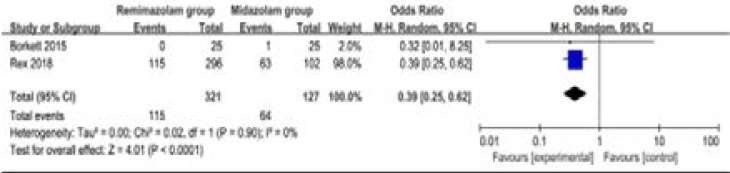
Forest plot for the meta-analysis of adverse events.

**Figure 9:**
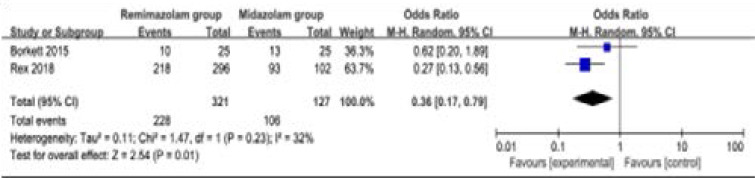


Compared to midazolam for gastrointestinal endoscopy, remimazolam was associated with lower need for rescue medication (very low quality, n=528; OR=0.09; 95% CI=0.01 to 0.80; P=0.03; Figure 4; three RCT included11, 14, 15), shorter total recall (low quality, n=107; Std. MD=0.93; 95% CI=0.15 to 1.72; P=0.02; Figure 5; two RCT included14, 15) and delayed recall (moderate quality, n=107; Std. MD=0.44; 95% CI=0.05 to 0.83; P=0.03; Figure 6; two RCT included14, 15), but showed no significant impact on fully alert (very low quality, n=425; Std. MD=-0.75; 95% CI=-1.58 to 0.08; P=0.08; Figure 7; two RCT included11, 15). The incidence of hypotension (moderate quality, n=448; OR=0.39; 95% CI=0.25 to 0.62; P<0.0001; Figure 8; two RCT included11, 15) and adverse events (low quality, n=448; OR=0.36; 95% CI=0.17 to 0.79; P=0.01; Figure 9; two RCT included11, 15) were found to be lower in remimazolam group than that in midazlam group.

## Discussion

Remimazolam is known as an ultrashort-acting benzodiazepine for the sedation of endoscopy through acting on GABA receptors^20^–^24^. Remimazolam is featured by rapid breakdown to inactive metabolites because of its carboxylic ester linkage. The mean terminal elimination half-life of remimazolam is 0.75 hours compared to 4.3 hours of midazolam^11^. Remimazolam was reported to provide adequate procedural sedation for endoscopy, and faster recovery than midazolam^14^, ^15^. Our meta-analysis suggests that remimazolam was associated with significantly higher procedure success, shorter total recall and delayed recall for gastrointestinal endoscopy than midazolam, indicating that remimazolam could result in better sedation and recovery than midazolam.

In one phase IIb study comparing the safety and efficacy of remimazolam and midazolam in patients undergoing colonoscopy, over 82.5% of the remimazolam patients obtained sufficient sedation after just the initial dose, compared with 46.3% of midazolam patients. The time to the procedure start was shorter in remimazolam group than midazolam group. Sedation with remimazolam led to an easier workflow compared with midazolam, which was consistent with less need of rescue sedative medication (2.5%–7.5%) in remimazolam than that in midazolam^14^. Lower need for rescue medication for remimazolam group was observed than that in midazolam group for gastrointestinal endoscopy based on the results of our meta-analysis.

The remimazolam 5.0/3.0 mg-dose group was revealed to have the higher efficacy rate and the better safety profile than remimazolam 8.0/3.0 mg and 7.0/2.0 mg group, which indicated that that this initial dose, combined with top-up doses of up to 3 mg may be ideal for the sedation of gastrointestinal endoscopy. There were few cases of adverse events (e.g. hypotension and bradycardia)^14^. Regarding the sensitivity analysis, significant heterogeneity remained, and there was no heterogeneity after excluding the study conducted by Borkett15. Two reasons may account for this heterogeneity. Firstly, initial dose of remimazolam (5.0 mg) in combination with another 2.5 mg or 3 mg may be better for the sedation than that of remimazolam 0.20 mg/kg. Secondly, the various procedures of gastrointestinal endoscopy and colonoscopy produced different pain intensity, and needed different levels of sedation.

Furthermore, the incidence of hypotension and adverse events in remimazolam group was lower compared to that in midazolam group for gastrointestinal endoscopy in our meta-analysis, suggesting the better safety of remimazolam than midazolam. The specific incidence of hypotension was about 35.8% in remimazolam group as compared to 50.4% midazolam group, while the incidence of adverse events were about 71% versus 83.5% between two groups. The adverse events mainly included hypotension, hypertension, bradycardia, tachycardia, hypoxia, nausea and vomiting etc^11^, ^15^.

This meta-analysis has several potential limitations that should be taken into account. First, our analysis is based on only three RCTs, and two of them have a small sample size (n<100). Overestimation of the treatment effect is more likely in smaller trials compared with larger samples. Next, there is significant heterogeneity in this meta-analysis, different methods of remimazolam and surgical procedures may have an influence on the pooling results. Finally, some unpublished and missing data may lead bias to the pooled effect.

## Conclusion

Remimazolam is better for the sedation for gastrointestinal endoscopy than midazolam with regard to higher procedure success, lower need for rescue medication, shorter total recall and delayed recall, as well as reduced adverse events.
